# Structures of the Ultra-High-Affinity Protein–Protein Complexes of Pyocins S2 and AP41 and Their Cognate Immunity Proteins from *Pseudomonas aeruginosa*

**DOI:** 10.1016/j.jmb.2015.07.014

**Published:** 2015-08-28

**Authors:** Amar Joshi, Rhys Grinter, Inokentijs Josts, Sabrina Chen, Justyna A. Wojdyla, Edward D. Lowe, Renata Kaminska, Connor Sharp, Laura McCaughey, Aleksander W. Roszak, Richard J. Cogdell, Olwyn Byron, Daniel Walker, Colin Kleanthous

**Affiliations:** 1 Department of Biochemistry, University of Oxford, South Parks Road, Oxford OX1 3QU, UK; 2 Institute of Infection, Immunity and Inflammation, College of Medical, Veterinary and Life Sciences, University of Glasgow, Glasgow G12 8QQ, UK; 3 WestCHEM, School of Chemistry, College of Science and Engineering, University of Glasgow, Glasgow G12 8QQ, UK; 4 Institute of Molecular Cell and Systems Biology, College of Medical, Veterinary and Life Sciences, University of Glasgow, Glasgow G12 8QQ, UK; 5 School of Life Sciences, College of Medical, Veterinary and Life Sciences, University of Glasgow, Glasgow G12 8QQ, UK

**Keywords:** EDTA, ethylenediaminetetraacetic acid, IPE, immunity protein exosite, PEG, polyethylene glycol, bacteriocin, *P aeruginosa*., pyocin S2, pyocin AP41, immunity protein

## Abstract

How ultra-high-affinity protein–protein interactions retain high specificity is still poorly understood. The interaction between colicin DNase domains and their inhibitory immunity (Im) proteins is an ultra-high-affinity interaction that is essential for the neutralisation of endogenous DNase catalytic activity and for protection against exogenous DNase bacteriocins. The colicin DNase–Im interaction is a model system for the study of high-affinity protein–protein interactions. However, despite the fact that closely related colicin-like bacteriocins are widely produced by Gram-negative bacteria, this interaction has only been studied using colicins from *Escherichia coli*. In this work, we present the first crystal structures of two pyocin DNase–Im complexes from *Pseudomonas aeruginosa*, pyocin S2 DNase–ImS2 and pyocin AP41 DNase–ImAP41. These structures represent divergent DNase–Im subfamilies and are important in extending our understanding of protein–protein interactions for this important class of high-affinity protein complex. A key finding of this work is that mutations within the immunity protein binding energy hotspot, helix III, are tolerated by complementary substitutions at the DNase–Immunity protein binding interface. Im helix III is strictly conserved in colicins where an Asp forms polar interactions with the DNase backbone. ImAP41 contains an Asp-to-Gly substitution in helix III and our structures show the role of a co-evolved substitution where Pro in DNase loop 4 occupies the volume vacated and removes the unfulfilled hydrogen bond. We observe the co-evolved mutations in other DNase–Immunity pairs that appear to underpin the split of this family into two distinct groups.

## Highlights

• We have identified two different bacteriocin DNase–Im subfamilies.
• First structures of pyocin DNase domains in complex with neutralising Im proteins.
• The subfamilies are characterised by distinct Im helix III motifs.
• ImAP41 lacks the key Asp in Im helix III and one of the conserved interfacial waters.
• New DNase–Im family expands the region that governs bacteriocin selectivity.

## Graphical abstract

**Figure:**
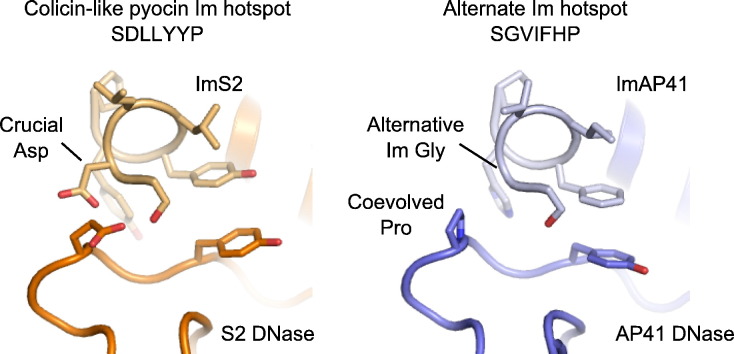

## Introduction

Specific interactions between proteins are key to their organisation into proteomes, with their number typically exceeding by orders of magnitude the number of genes within a genome [1]. Specificity in protein–protein interactions is essential in the normal development of vertebrate immune systems [2,3] and tissues. They are also indispensable for invasion and subversion of host cells by bacteria and viruses [4]. However, the biophysical and structural basis for specific recognition of one protein over others is still largely focused on just a few model systems. One of the best understood and widely studied specificity systems in protein–protein recognition is the interaction of colicin endonucleases (DNases) with their immunity (Im) proteins [5]. DNase colicins are potent bacteriocins (protein antibiotics) that kill *Escherichia coli* during intraspecies competition for resources. Immunity proteins protect the colicin producing, or “colicinogenic”, bacteria by binding and inactivating colicin nucleases with very high affinity (*K*_d_ ≈ 10^− 14^ M) and specificity [6,7]; the discrimination in binding affinity between cognate and non-cognate nucleases by Im proteins can be up to 10 orders of magnitude [8]. A large body of structural and biophysical data on cognate and non-cognate colicin DNase–Im protein complexes has been generated [7–10] and was recently reviewed by Papadakos *et al.* [11]. The wealth of data has served as an impetus for the exploitation of the system in protein design and directed evolution approaches [12–15]. However, to date, our understanding of how these complexes achieve such high affinity and specificity is based entirely on a closely related branch of this DNase–Im protein superfamily, the colicins. Nuclease bacteriocin–Im pairs are widespread amongst Gram-negative bacteria and are present in clinically important *Pseudomonas spp*. and *Klebsiella spp*. [16]. The present work sets out to determine the structure of the pyocin DNase domains of PyoS2 and PyoAP41 in complex with their cognate immunity protein in order to sample the structural diversity of protein–protein interactions between bacteriocin–Im pairs.

The DNase colicin family (ColE2, ColE7, ColE8 and ColE9) has a domain structure that is representative of all the DNase bacteriocins. In addition to their DNase domain, these proteins possess domains for binding to outer membrane receptors and periplasmic translocation proteins [11]. These domains together expedite entry to the cytoplasm of a conserved 15-kDa C-terminal cytotoxic DNase domain, the catalytic core of which is the ubiquitous ≈ 30-amino-acid HNH/ββα-Me motif. HNH motif nucleases are found in all three kingdoms of life where they serve a variety of functions, including homologous recombination, DNA repair and apoptosis [17]. In the context of DNase bacteriocins, the HNH motif, which coordinates a single divalent metal ion within its centre, binds to the minor groove of genomic DNA causing random cleavages that overwhelm the DNA repair machinery of the cell and result in death within minutes of intoxication [18,19].

The immunity protein associated with DNase colicins has an unusual mode of action; rather than binding directly to the DNase catalytic core, these proteins associate with the nuclease domain at an exosite adjacent to the enzyme active site. This association blocks substrate DNA binding but leaves the catalytic residues of the HNH motif completely exposed [20–22]. A consequence of exosite binding is that it allows for extensive sequence diversification of residues at the DNase–Im protein binding interface, without alteration of residues that contribute to catalytic activity. As a result, the protein–protein interface of colicin DNase–Im protein complexes exhibits a much larger level of sequence diversity compared with the rest of the protein [5].

Previous work has established that a dual-recognition binding mechanism underpins Im protein specificity for colicin DNases whereby a small, conserved α-helix (helix III) provides the bulk of the binding free energy and an adjacent larger α-helix (helix II) largely governs the interaction specificity of the Im protein [23]. The high degree of discrimination in colicin DNase–Im protein pairs comes from chemical frustration at the protein–protein interface when residues within non-cognate pairings are forced to dock into inappropriate binding pockets on the enzyme surface [8,24,25]. In addition, recent directed evolution and structural studies on colicin DNase–Im protein complexes have also highlighted the importance of interhelical loops as having both direct and indirect roles in sculpting Im protein specificity [12,25], emphasising that specificity in this system is multi-faceted and highly cooperative.

An area of colicin DNase–Im protein specificity that has yet to be explored is the conserved Im helix III, which constitutes a binding energy hotspot on the hypervariable surface of the DNase domain. Whilst all the colicin DNase–Im complexes studied are conserved within helix III, the cognate Im proteins for a number of *Pseudomonas spp*. homologues of colicin DNases (pyocins) are divergent within this conserved binding epitope. To assess the ramifications of this divergence, we set out to determine the structures of the DNase–Im protein complexes from pyocin S2 and AP41, which possess a “classical” colicin-like and divergent helix III, respectively.

In this work, we present the first crystal structures of bacteriocin DNase–Im complexes from *Pseudomonas aeruginosa*. Comparison of these complexes yields new insights into the stability and specificity of ultra-high-affinity protein–protein interactions and demonstrates the structural consequences of sequence divergence at Im helix III, the binding energy hotspot.

## Results and Discussion

### Bioinformatics analysis of the γ-proteobacteria bacteriocin DNase–Im protein pairs

**Fig. 1:**
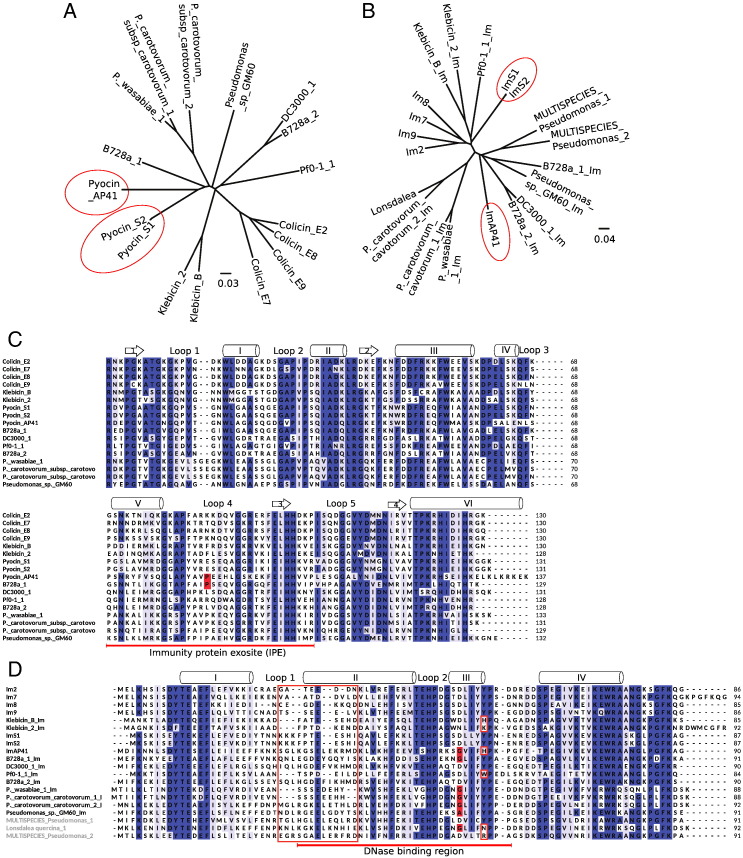
Sequence analysis of bacteriocin HNH/ββα-Me DNase domains and their immunity proteins. Phylogenetic trees generated for bacteriocin HNH DNase domains (A) and immunity proteins (B) from the sequence alignments [(C) and (D), respectively]. (A and B) Pyocins S2 and AP41 are highlighted in the phylogenetic trees. The PyoS2 and PyoAP41 DNase domains separate in a similar manner whilst their respective Im proteins are in disparate clades. (C and D) Sequence alignments are coloured by conservation (dark blue, identical; pale blue, highly similar and annotated with secondary structure elements shown above the sequence); arrows and cylinders represent strands and helices, respectively. Interprotein interacting regions are indicated (red underline). Im loop 1 is of varying length. There is a distinct sequence divergence within the conserved Im helix III where glycine substitutions correlate with a proline mutation within the DNase domain (red boxes).

Sequence analysis was performed for 17 bacteriocin DNase–Im pairs from a diverse subset of γ-proteobacteria. The DNase domains share considerable sequence identity (40–99%) across all species and phylogenetic analysis shows that they can be separated into groups that correspond to the producing species (Fig. 1a). Sequence identity is found throughout the nuclease domains; however, considerable sequence divergence is observed in DNase helix V and loop 4, which has been shown structurally and biochemically to be the immunity protein exosite (IPE) (Fig. 1a and c). Overall, there is less identity between the corresponding Im proteins, with identity ranging from 30% to 90% (Fig. 1b and d). In particular, the region from the middle of Im helix I to the end of helix II has very low conservation and the loop between these secondary structure elements (loop 1) is of varying length. These regions have been shown previously to be important in determining specificity for the colicin DNase–Im complexes [12,25]. The N-terminal portion of helix I, the region covering loop 2 and helix III (which constitutes the binding energy hotspot in all Im proteins), and helix IV shows much greater sequence conservation. Sequence analysis of colicins has revealed that regions under positive selection are within both the cytotoxic domain of the colicins and their immunity proteins [26]. Using omegaMap [27], we have mapped these at codon resolution and found that regions under positive selection lie within the DNase IPE (Supplemental Fig. 1) and Im helix II (data not shown). An equivalent analysis for pyocins also predicted the DNase IPE to be under positive selection although at lower probability than was predicted for the colicins DNases (Supplemental Fig. 1). Overall, the lower probability may be due to increased recombination rates as pyocins are more prevalent in the environment [28].

Whilst most of the Im proteins segregate in a similar manner to their respective DNases, ImAP41 is phylogenetically different (Fig. 1C). The DNase domains of pyocins S2 and AP41 share 60% sequence identity and their close relationship is reflected in the phylogenies (Fig. 1A) [29]; however, ImS2 and ImAP41 unexpectedly separate into different clades (Fig. 1B). ImS2 has greater sequence similarity to the colicin immunity proteins whilst ImAP41 is a member of a distinct clade of Im proteins. A feature that distinguishes between the clades can be seen in Im helix III, the binding energy hotspot. In ImS2, the DNase binding hotspot residues are almost identical to those in colicin Im proteins (SDLIYYP) whilst ImAP41 represents a group with an alternate hotspot sequence in which the Asp (the most critical residue in colicin Im proteins) is replaced by Gly [24,30]. Im helix III forms the main stabilising interactions with the DNase domain, and thus, changes in this key helix are likely to generate different specificities and potentially alter the binding modes of Im proteins. To investigate this in detail, we set out to determine the structural basis for specificity by comparing and contrasting canonical and non-canonical pyocin DNase–Im complexes.

### The crystal structure of AP41 DNase domain

**Fig. 2:**
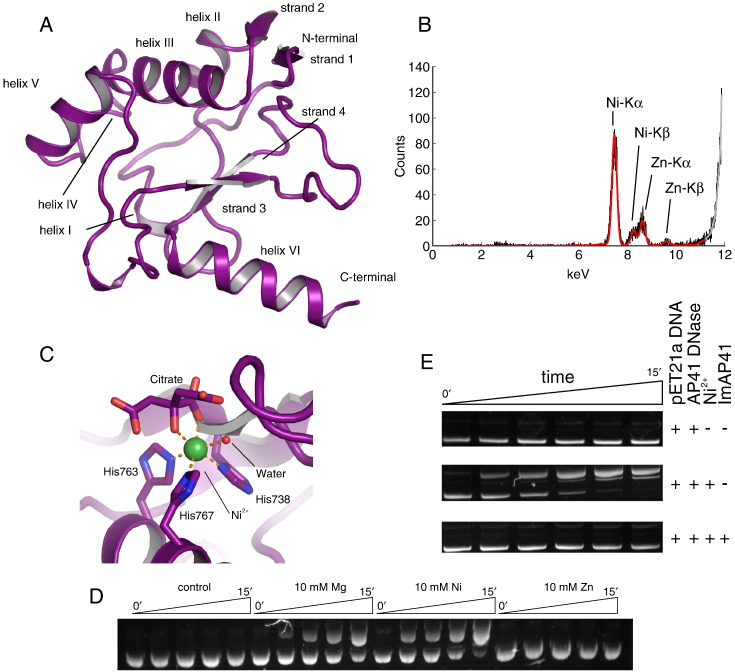
Structure of pyocin AP41 DNase domain. (A) Crystal structure of AP41 DNase domain shown in cartoon representation (carbon atoms coloured purple). The AP41 DNase domain has mixed α/β secondary structure that is typical of the bacteriocin DNase fold. (B) Raw (black) and fitted (red) X-ray fluorescence spectra of crystalline AP41 DNase domain showing that the dominant metal in the AP41 DNase domain is nickel with a small amount of zinc. (C) Details of the Ni^2 +^ coordination in the AP41 DNase domain. AP41 DNase domain is shown in cartoon representation with selected side chains shown as sticks. The Ni^2 +^ is a green sphere and water atom is a red sphere. Carbon atoms are coloured purple, oxygen atoms are coloured red and nitrogen atoms are coloured blue. Metal coordination is depicted by orange broken lines. (D) Plasmid nicking assay showing that AP41 DNase is active in the presence of both Ni^2 +^ and Mg^2 +^ ions whilst Zn^2 +^ does not support catalytic activity. (E) Plasmid nicking assay showing that EDTA-treated AP41 DNase domain is inactive. AP41 DNase is active in the presence of Ni^2 +^ causing single-stranded hydrolysis of the plasmid template that is prevented by ImAP41.

We first determined the crystal structure of pyocin AP41 DNase in isolation to (i) verify that pyocin DNases adopt the HNH/ββα-Me fold and (ii) establish if Im binding causes any structural changes in the protein. Pyocin AP41 DNase domain protein crystals diffracted X-rays to 1.5 Å resolution and were found to belong to the *P*4_1_2_1_2 space group (Table 1). The structure was solved by molecular replacement and refined to final *R*_free_/*R*_work_ = 0.197/0.173 with good stereochemistry (Fig. 2A and Table 1).

AP41 DNase domain adopts the typical mixed α/β HNH fold seen in other DNase bacteriocins. This fold is essentially identical to that of the colicin E9 and E2 DNases with RMSD values of 0.83 Å and 0.63 Å, respectively, for C^α^ atoms. Interestingly, AP41 DNase possesses a positively charged C-terminal extension (LKRKEK) that is not present in other nuclease domains. As with the archetypal ColE9 structure, the HNH/ββα-Me motif resembles a zinc finger where three histidine side chains coordinate a metal ion. We identified the metal in the structure by X-ray fluorescence spectroscopy of the crystalline sample. The spectrum showed that most of the metal ions in the crystal were Ni^2 +^ but there was a small amount of Zn^2 +^ (Fig. 2B). In the AP41 DNase structure, the Ni^2 +^ ion displays octahedral coordination geometry, which is coordinated by His738, His763 and His767; by a single water molecule from solution; and by the hydroxyl and a carbonyl oxygen from a citrate anion within the crystallisation solution (Fig. 2C). The citrate provides the equivalent coordination that is provided by the phosphate anion present in DNA bound forms of colicin DNases [22,31].

The presence of Ni^2 +^ in the AP41 DNase active site is consistent with the first-solved HNH colicin structure (for ColE9) in which Ni^2 +^ was also bound [20]. However, it was later shown that many transition and alkaline earth metals can be coordinated in the active site [19,32]. We found that the AP41 DNase is most active in the presence of Ni^2 +^ or Mg^2 +^ ions whilst showing no detectable activity in the presence of Zn^2 +^ (Fig. 2D). We have also confirmed that the DNase activity is inhibited by its immunity protein, ImAP41 (Fig. 2E).

### The crystal structures of the pyocins AP41 DNase–ImAP41 and S2 DNase–ImS2 complexes

**Fig. 3:**
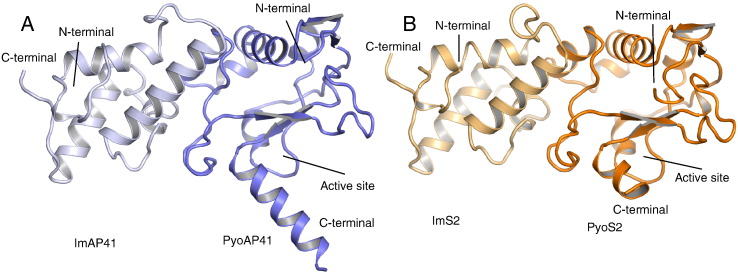
Crystal structure of pyocin DNase–Immunity protein complexes. Identical views of the pyocin AP41 DNase–ImAP41 (A) and pyocin S2 DNase–ImS2 (B) complexes shown in cartoon representation. AP41 DNase and Im coloured blue and light blue, respectively; S2 DNase and Im coloured orange and light orange, respectively. In both complexes, the DNase domains adopt the typical mixed α/β DNase fold whilst the Im is a four-helix bundle.

Pyocin DNase–Im complexes were crystallised and structures solved to 2.0 Å and 1.8 Å for the pyocin AP41 DNase–ImAP41 and pyocin S2 DNase–ImS2 complexes, respectively (Table 1 and Fig. 3A and B). The DNase domain in both complexes adopts the typical HNH/ββα-Me DNase fold. Superposition of the AP41 DNase structures revealed that they are almost identical (RMSD = 0.64 Å for C^α^ atoms); however, there is a slight change in the angle of the C-terminal helix that is likely due to crystal packing. The altered C-terminal helix repositions the histidines such that they can no longer coordinate a metal ion seen in the pyocin AP41 DNase structure. The structures of the pyocin AP41 and S2 DNase domains in complex with their cognate Im proteins are almost identical (1.26 Å C^α^ RMSD) and reveal similarities to the previously solved DNase–Im structures. The Im is a four-helix bundle that is bound at an exosite, adjacent to the DNase active site (Fig. 3A and B). However, the longer C-terminal helix seen in AP41 is not present in S2 or indeed in other bacteriocin DNase domains.

**Fig. 4:**
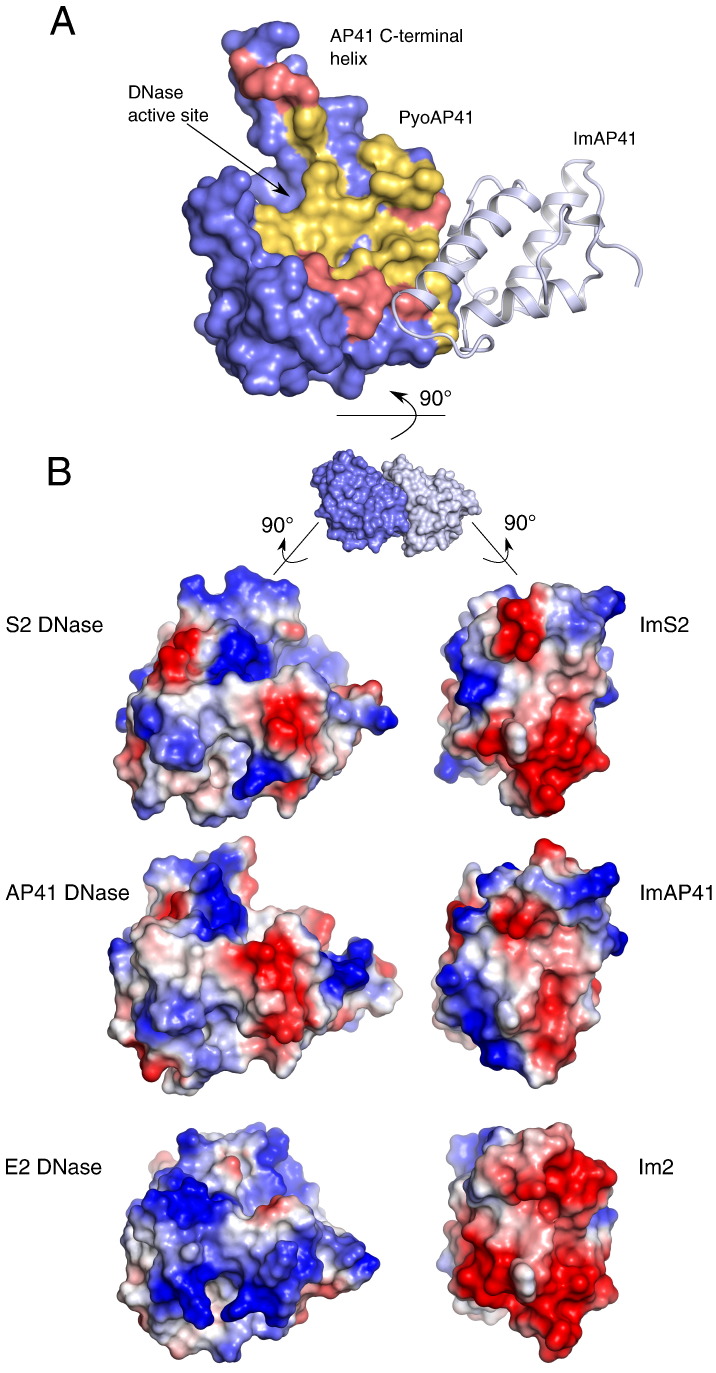
Surface analysis of pyocin DNase–Im protein complexes. (A) AP41 DNase domain represented as a blue surface and ImAP41 shown in cartoon representation. Residues conserved between pyocins and colicins in the DNA binding groove are coloured yellow. Residues differing from the colicin DNA binding groove are coloured pink and located in the periphery of the DNA binding region. (B) The electrostatics of DNase and Im proteins from the PyoS2, PyoAP41 and ColE2 DNase–Im complexes (red negative charge; blue positive charge). The DNase domains and their respective Im proteins have complementary charge distributions. However, the colicin E2 DNase–Im2 interface is significantly more polar whilst the AP41–ImAP41 interprotein interface is composed of fewer charged residues.

The structures of the colicins ColE7 and ColE9 bound to the minor groove of double-stranded DNA have been reported [22,31]. The DNA binding region comprises residues from the majority of the secondary structure elements within the DNase domain. The contacts with DNA are formed by residues lining a concave surface (Fig. 4A) that contains the active-site histidines. A large number of residues in the DNA binding region are common between colicins and pyocins, indicating that pyocins are likely to share the non-specific nuclease activity of colicins (Figs. 2D and 4A, yellow). However, the pyocins have some sequence divergence when compared with the colicins (Fig. 4A, pink). The structures of colicins bound to DNA reveals contacts to the C-terminal α-helix. Given the positively charged nature of the extended AP41 C-terminal helix, it is possible that it forms additional contacts with its substrate DNA; however, additional structural or biochemical information would be required to confirm this.

### Overview of the DNase–Im interaction in pyocins

The secondary structure elements involved in the colicin DNase–Im complex interactions have been determined from previous crystal structures [20,33]. Helix II and helix III from the Im interact with the IPE of the DNase that comprise a contiguous amino acid sequence encompassing helix V and loop 4 (Fig. 1C). The position of the Im is not always the same as in colicin–Im complexes. A 19° rotation is observed between the position of the Im when the ColE7–Im7 structure is compared with the structures of ColE9–Im9 and ColE2–Im2 [23]. Our structures of pyocin DNase–Im complexes do not exhibit any significant degree of rotation when compared with the ColE9–Im9 structure. These structures also show that colicin DNase–Im binding interfaces display similar degrees of shape and charge complementarity (Table 2 and Fig. 4B).

**Fig. 5:**
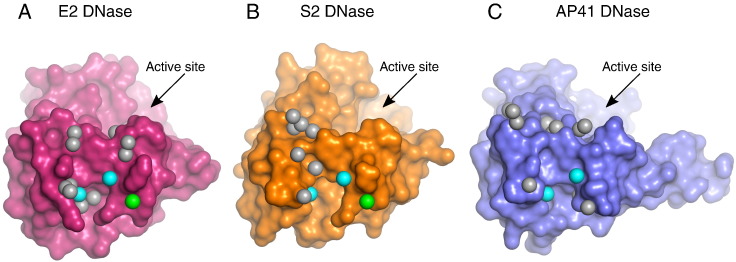
Interfacial waters in DNase–Im complexes. Details of the interfacial water molecules for (A) colicin E2 DNase, (B) pyocin S2 DNase and (C) pyocin AP41 DNase at the DNase–Im interface. DNase domains are shown in surface representation, and water molecules are shown as spheres. Water molecules conserved across all three structures are coloured cyan. The water molecule associated with the conserved hotspot aspartate of the Im proteins is coloured green. All other water molecules are coloured grey. The three interprotein interfaces contain similar numbers of buried water molecules (see Table 2).

In addition to direct polar contacts, water-mediated intermolecular interactions are essential for high-affinity colicin DNase–Im complex formation. The high-resolution structure of ColE2–Im2 identified numerous ordered solvent molecules; however, only three were shown to be conserved between other colicin DNase–Im complexes (Fig. 5A) [33]. Extending this analysis to include the pyocins reveals that all the complexes have a similar number of interfacial water molecules (Fig. 5 and Table 2). All three conserved water molecules are observed in the pyocin S2 DNase–ImS2 structure (Fig. 5B). However, pyocin AP41 shares only two of the conserved water molecules; the coordination of the third water is specific to the colicin helix III hotspot (see below) (Fig. 5C and Table 2).

### The hypervariable DNase IPE has a conserved structure

**Fig. 6:**
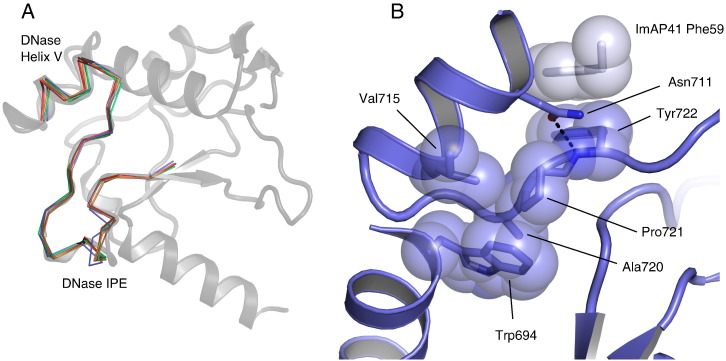
The DNase IPE is an extended loop that adopts a conserved structure. (A) Cartoon representation of the DNase domain of AP41 with C^α^ traces of the DNase IPE for ColE2, ColE7, ColE9, PyoS2 and PyoAP41 in complex with their cognate Im—data not shown here. The IPE does not adopt any regular secondary structure but the different sequences superpose well (0.56 Å RMSD for backbone atoms). (B) Cartoon representation of pyocin AP41 DNase domain (blue) with selected side chains shown as sticks and transparent space-filling spheres. Conserved amino acids in the IPE stabilise Pro721 that bridges the hydrophobic core of the enzyme (Trp694) to the Im binding site. In all DNase–Im complex structures, the residue following Pro721 makes contacts with the Im hotspot aromatic residues.

The IPE is largely devoid of regular secondary structure and is poorly conserved in sequence across colicins and pyocins (37% of the IPE is well conserved over a total of 27 residues). However, there is a very high degree of structural conservation of backbone atoms: the extended loops superpose with an RMSD of 0.56 Å (backbone atoms; Fig. 6a). Within the IPE, conserved residues maintain the structure of the loop, and the side chain of the conserved Pro (PyoAP41 Pro721) is anchored in the hydrophobic core of the enzyme, packing against Trp694. PyoAP41 Pro721 is itself surrounded by other well-conserved amino acids—Asn711, Val715 and Ala720—and is stabilised by the side chain of Asn711 (Fig. 6b) These contacts restrict the motion of Pro721 and Tyr722 in PyoAP41 whilst the conserved Gly718 is required to adopt this conformation. The structure of the IPE is underpinned by an NxxxφxxGxAP motif (where φ represents A, I, L or M) whilst two conserved peptide hydrogen bonds (Phe722–Pro710 and Glu733–Ala723) further stabilise its conformation. The NxxxφxxGxAP motif stabilises Tyr722 that forms a parallel π-stacking interaction with ImAP41 Phe59 from the helix III hotspot (Fig. 6b). The conserved sequence and position of the NxxxφxxGxAP motif reveal it to be an important structural feature of the otherwise highly variable IPE. Strikingly, the NxxxφxxGxAP motif has a low probability of positive selection whilst the rest of the IPE is under positive selection pressure (Supplemental Fig. 1).

### DNase–Im specificity determinants

**Fig. 7:**
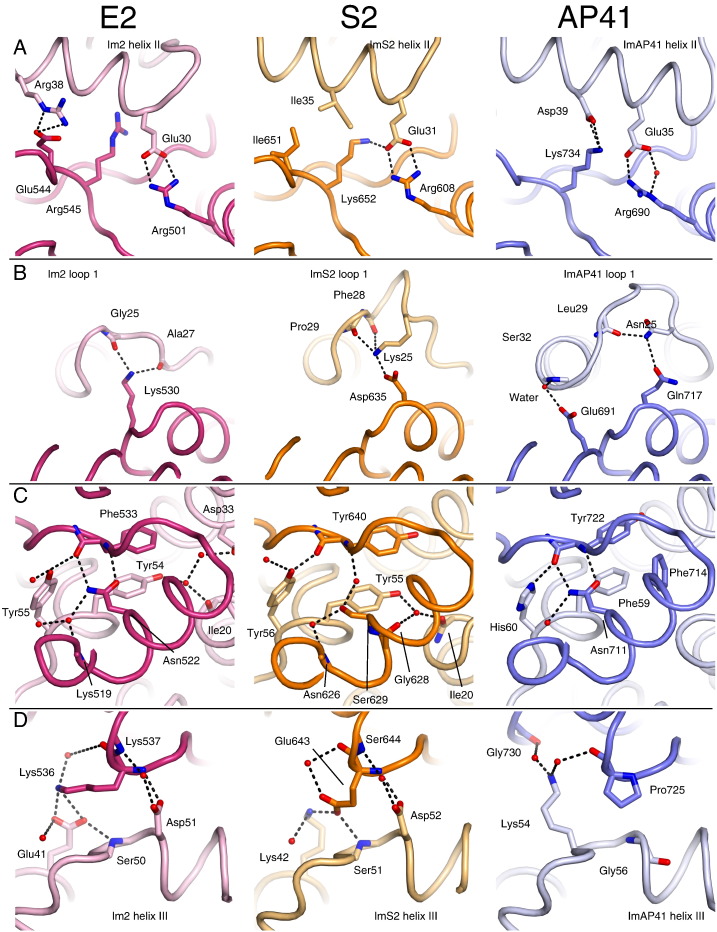
Details of the intermolecular pyocin DNase–Im interactions. Views of the specificity interactions between DNase and Im proteins, focusing on Im helix II (A and B) and helix III (C and D). Carbon atoms are coloured pink and light pink for E2, orange and light orange for S2 and blue and light blue for AP41 (immunity proteins are a lighter shade). Oxygen atoms are coloured red and nitrogen atoms are coloured blue. Key water molecules are shown as red spheres. Hydrogen bonds are shown as black broken lines.

Specific amino acids within the Im protein that contribute to binding DNase domains are found within a contiguous region of the protein. Specificity determinants within the Im proteins are largely located in the poorly conserved region from Im loop 1 to the end of helix II, whilst helix III is a conserved binding epitope for colicins (Fig. 1D). Within the region with low sequence identity, there is a striking feature; Im helix II contains a strictly conserved Glu (Glu30 in Im2). This forms a conserved intermolecular salt bridge to colicin E2 Arg501 and is seen in all DNase–Im structures (Fig. 7A). Im helix II is involved in a large number of specific interactions and poor packing in this region is the main determinant of low affinity in non-cognate complexes (Fig. 7A) [8,25].

Directed evolution experiments have shown that loop 1 is important in extending the specificity of Im proteins to other DNases [12]. Mutations here relieve packing defects caused by imperfect side-chain docking and represent a primary step in developing new specificity [25]. Consistent with a role in specificity, this region is poorly conserved. Loop 1 is not only a region with a low level of sequence identity but has numerous insertions (Fig. 1D). Compared with the colicin immunity proteins, ImAP41 loop 1 contains three additional amino acids and this is reflected in the more elaborate conformation it adopts (Fig. 7B, right). The increase in potential conformational flexibility of AP41 loop 1 is restrained by two additional intermolecular interactions (Fig. 7B, right). ImS2 loop 1 has a single amino acid insertion compared with ColE2 and adopts a stabilised conformation that also traps four water molecules (Fig. 7B). Despite these differences in loop 1, helix II from ImS2, Im2 and Im9 superpose very well whilst ImAP41 helix II is offset by ≈ 2 Å.

### Pyocin–Im hotspot intermolecular interactions

The kinetics and thermodynamics of Im binding to cognate and non-cognate colicin DNase domains has been studied in great depth [11]. Both cognate and non-cognate complexes display very fast association kinetics that are electrostatically driven. Once the initial complex is formed, rigid-body rotations and side-chain rearrangements are required for the high-affinity interactions seen in cognate complexes. The binding energy hotspot that encompasses Im loop 2 and helix III provides the main stabilising interaction. Non-cognate Im proteins have association kinetics similar to those of cognate complexes implying that the same underlying binding mechanism drives complex formation. However, the lower affinity of these complexes (*K*_d_ > 10^− 8^ M) is due to the fast off rate that results from the poor packing of helix II [9,24]. We propose that this dual-recognition binding mode is extended to all Im proteins, as the IPE of the DNase domains is structurally conserved.

The interactions of the two aromatic amino acids in the ImS2 hotspot are almost identical to those in Im2 and Im9, as might be expected from a high degree of primary sequence conservation (Fig. 7C). The aromatic amino acids in the ImAP41 hotspot (Phe and His) are of similar size to those in the colicin hotspot motif but have different chemical properties. The first aromatic residue, Phe59, forms a parallel π-stacking interaction with Tyr722 within pyocin AP41 DNase domain in a manner similar to the Tyr in Im2 and ImS2 (Fig. 7C). However, the polar environment of this side chain seen in Im2 and ImS2 is absent in AP41 and waters are excluded from this region. The PyoAP41–ImAP41 complex is stabilised by an orthogonal (T-shaped) π-stacking interaction between ImAP41 Phe59 and PyoAP41 Phe714. The second aromatic residue, His60, forms a hydrogen bond to the backbone carbonyl of Tyr722 making contacts equivalent to those of Im2 Tyr55 in the ColE2–Im2 complex and ImS2 Tyr56 in the PyoS2–ImS2 complex (Fig. 7C).

Although ImAP41 represents a different clade of Im, the presence of aromatic residues in identical positions within the motif strongly implies that the interaction between the DNase and all Im proteins can be described by a common binding mode, namely, the requirement for the exclusion of bulk water from this interface whilst retaining the interstitial waters and stacking of aromatic amino acids. Formation of DNase–Im contacts are diffusion limited and focused upon Im helix III [7]. Other Im protein regions stabilise the interaction through specific contacts and are required for high affinity. As the colicin Im helix III is well conserved, the effect of specificity determinants in helix III was not considered; however, our structures show that selectivity occurs within the Im hotspot. The colicin Im hotspot motif is [S/T]DLIYYP for the immunity proteins of colicins E2, E7, E8 and E9. In cognate and non-cognate complexes, the serine and aspartate residues within the motif form intermolecular interactions: Im2 Asp51 forms one direct and one water-mediated hydrogen bond to backbone amides in the DNase domain whilst the serine backbone amide is part of an intermolecular hydrogen bond network (Fig. 7D, left). Overall, the pattern of intermolecular interactions for the equivalent amino acids in the PyoS2–ImS2 interface is similar to that of ColE2–Im2 (Fig. 7D, middle). In the alternate hotspot, represented by PyoAP41–ImAP41, the invariant Asp is replaced by a Gly that has very different physicochemical properties. A complementary substitution in the DNase, PyoAP41 Pro725, accommodates the vacated space in ImAP41 (Fig. 7D, right). Furthermore, the cyclic pyrrolidine removes an amide that would leave an unfulfilled hydrogen bond. Interestingly, this reciprocal proline/glycine substitution is seen in all DNase sequences where Im proteins have alternate hotspots (Fig. 1D). The intercalation of side chains seen in the alternate hotspot would make a complex with a colicin-type hotspot extremely unfavourable due to the steric clashes that would occur with the larger amino acid side chains.

### Conserved waters at the DNase–Im interface

**Fig. 8:**
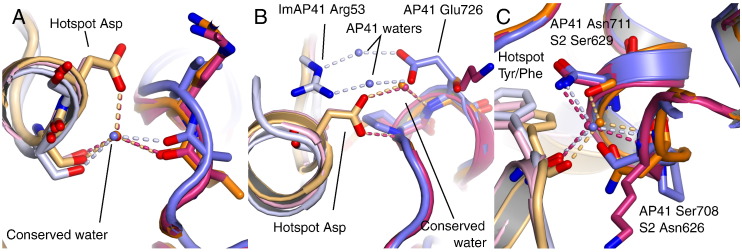
Conservation of water molecules in the DNase–Im interface. Conserved water molecules at the DNase–Im interface are shown with details of the molecular interactions. Proteins are shown in cartoon representation with selected side chains shown as sticks and coloured by carbon atom (colicin E2 DNase–Im2, pink/light pink; pyocin S2 DNase–ImS2, orange/light orange; pyocin AP41–ImAP41, blue/light blue). Water molecules are shown as spheres labelled by structure and hydrogen bonds are coloured by structure and shown as broken lines (orange for ColE2–Im2, red for PyoS2–ImS2 and light blue for PyoAP41–ImAP41). Oxygen atoms are coloured red and nitrogen atoms are coloured blue.

Bacteriocin DNase–Im interfaces contain numerous interstitial water molecules. Many water molecules confer stability whilst others provide specificity to the complexes. Three waters are strictly conserved in the ColE2–Im2, ColE7–Im7 and ColE9–Im9 complexes and have been proposed to act as pivots about which rigid-body movements could occur (ColE2–Im2: Fig. 6A) [34]. The Asp within the Im2 hotspot is involved in hydrogen bonds to two of these water molecules. The first water is coordinated by the side chains of Im2 hotspot Asp51 and Ser50 and a backbone carbonyl from DNase loop 4 (Fig. 8A). The second conserved interaction between the hotspot Asp51 and water is the water-mediated intermolecular hydrogen bond mentioned above (Figs. 6D, left, and 8B). Asp51 forms a polar contact to backbone amides within DNase loop 4 via water-mediated hydrogen bonds. The third water is coordinated by the backbone amides of Im2 Tyr54 and ColE9 Lys519 with a third hydrogen bond to the side chain of ColE2 Asn522 (Fig. 8C). ImS2 shares the conserved waters with colicins and the interactions observed are analogous for all three conserved water molecules (Fig. 8A–C). As ImAP41 lacks the acidic Asp amino acid within its hotspot, the interactions with water molecules are different. Nevertheless, two of the conserved water molecules are observed at the AP41 interprotein interface. Surprisingly, the first water molecule is in an almost identical position compared to the E2 and S2 complex structures, even though the Asp within the motif is absent. Coordination of this water is maintained by the ImAP41 Ser55 side chain and PyoAP41 Ala723 carbonyl that are equivalent to the interactions seen in the ColE2–Im2 (Fig. 8A). However, the second water that is coordinated by the Asp side chain in Im2 and ImS2 is absent in the AP41 complex (Fig. 8B); the absence of the acidic side chain from ImAP41 removes this water-mediated interaction. This difference between the colicin hotspot sequence and the alternative sequence indicates an additional mechanism through which specificity is attained. The cognate AP41 complex is strengthened by two water-mediated intermolecular hydrogen bonds between the guanidinium group of ImAP41 Arg53 and the carboxylic acid of Glu726, the side chain of which occupies the position of the water in Im2 and ImS2 (Fig. 8B). The third conserved water is common in the three structures analysed (Fig. 8C). The two conserved water molecules that are seen in the pyocin AP41 DNase–Im complex are also observed in the apo AP41 DNase structure.

### Concluding remarks

In this work, we have solved the first structures of the DNase domains of pyocins S2 and AP41 in complex with their cognate Im proteins, in addition to the structure of the isolated pyocin AP41 DNase domain. Our structures reveal that, in a similar manner to the colicins, binding surfaces of the DNase domains and cognate immunity proteins possess complimentary shapes and charges with interfacial water molecules mediating important intermolecular hydrogen bonds. However, these structures also reveal a distinct divergence from the colicin canonical DNase–Im interface. Our bioinformatics search identifies a distinct family of Im proteins where the sequence of the key helix III is different from the identified sequence in colicin specific Im proteins (Fig. 1b and d). However, surprisingly, the relative domain positions in the AP41 DNase–Im complex superimpose well with the colicin E9 DNase–Im9 complex and do not exhibit the twisted relative domain positioning that is observed in the ColE7–Im7 complex [23].

The structure of the AP41 DNase–Im complex reveals that specificity is still largely governed by Im helix II as this contains the vast majority of specificity determining contacts. However, the specific contacts made between the DNase domain and helix III of ImAP41 are representative of a distinct subfamily of Im proteins. This alternative hotspot motif is found within a number of other sequences, where the Gly seen in ImAP41 is replaced with Ala in one instance. Importantly, the alternate Im hotspot is always paired with a co-evolved Pro in the DNase domain. This branch is a distinct family of DNase–Im complexes where the formation of non-cognate complexes with canonical Im helix III proteins would be very unfavourable.

Our analysis shows that Im loop 2, the loop that precedes helix III, contains more mutations in Im proteins that have the alternate helix III motif. These co-evolved mutations are likely to facilitate the alternate helix III motif or provide additional stabilising intermolecular interactions, as seen with ImAP41 Arg53. Im loop 1 also exhibits large variations between different Im proteins. Whilst this loop forms intermolecular interactions with the DNase domain, they largely involve the backbone and thus sequence variation is tolerated. It is well documented that mutations in loop 1 facilitate further mutations that would otherwise be unfavourable due to packing frustrations. Our observations from the pyocin AP41 DNase–ImAP41 structure may be naturally occurring examples of this recent discovery.

## Materials and Methods

### Bioinformatics

Colicin nuclease domain and immunity protein homologues were identified using a BLAST search of the National Center for Biotechnology Information non-redundant protein database. A number of homologues from representative organisms were aligned using the ClustalW algorithm [35]. Bootstrapped phylogenetic trees were then created using the nearest-neighbour joining method.

Positive selection was assessed using the program omegaMap [27]. Fifty colicin or pyocin DNase sequences and their respective Im were aligned and parameters were derived using a hidden Markov model over 250,000 iterations. The colicin DNase dataset had 80% sequence identity in the alignment; however, sequence identity for the pyocins was bimodally distributed at either > 95% or ≈ 60%. Prior parameter distributions were modified from Ref. [36], assuming no positive selection was present. Indels in the pyocin Im proteins resulted in poor alignments and hindered positive selection probability analysis.

### Expression of pyocins S2 and AP41

The full-length PyoS2 and the pyocin AP41 nuclease domain (residues 641–777) were expressed and purified in complex with their full-length immunity proteins in *E*. *coli* BL21 (DE3) cells harbouring a pET21a-derived expression vector containing the pyocin operon with a C-terminal 6 × His tag fused to the immunity protein. Cells were grown at 37 °C until OD_600 nm_ = 0.6–0.8 and protein production was induced with 1 mM isopropyl β-d-1-thiogalactopyranoside. Cultures were grown for a further 4 h at 37 °C and harvested by centrifugation. Cells were lysed by sonication and proteins purified by nickel affinity chromatography and size-exclusion chromatography as previously described for nuclease colicins [32,37]. AP41 DNase domain was isolated by disrupting the AP41 DNase–ImAP41 complex using 6 M guanidine hydrochloride and retaining ImAP41 on nickel affinity resin. Proteins were further purified by size-exclusion chromatography in a buffer containing 150 mM NaCl and 50 mM Tris (pH 7.5) (AP41 DNase and the AP41 DNase–ImAP41 complex) or in a buffer containing 200 mM NaCl and 50 mM Tris (pH 7.5) (PyoS2–ImS2 complex).

### Crystal structure determination of the pyocin S2 DNase–ImS2 complex

Crystallisation screens for the intact PyoS2–ImS2 complex were performed in the presence of chymotrypsin at a 1:100 ratio of protease to pyocin. Small rod-shaped crystals (50–100 μm) formed in 0.2 M sodium bromide, 20% (w/v) polyethylene glycol (PEG) 3350 and 0.1 M 2-[bis(2-hydroxyethyl)amino]-2-(hydroxymethyl)propane-1,3-diol (Bis-tris) propane (pH 6.5) after approximately 1 month at 20 °C. Crystals were cryoprotected by increasing the PEG 3350 concentration to 35% (w/v) and data were collected at beamline I04 of Diamond Light Source (United Kingdom) at 100 K. Crystals were of the space group *P*2_1_ and diffracted to 4.2 Å resolution; however, the structure could not be solved by molecular replacement or experimental phasing using the bromide anomalous signal. Optimisation trays were prepared, and when crystals failed to form after 2 months at 20 °C, the screens were transferred to 4 °C. Crystals formed after an additional 4 months under the same conditions and were cryoprotected as described above. Diffraction data were collected from a single cryocooled crystal at beamline I03 of Diamond Light Source (United Kingdom). Crystals were of the space group *P*2_1_2_1_2_1_ and diffracted to 1.63 Å (final data were processed to 1.8 Å resolution). Diffraction data were integrated using XDS [38] and scaled using Aimless [39] from the CCP4 suite [40].

As the specific fragment of PyoS2 present in the crystal was unknown, molecular replacement with various fragments of ColE2 and its immunity protein was attempted using Phaser [41]. The asymmetric unit was found to contain four copies of the pyocin S2 DNase–ImS2 complex, with the entirety of the immunity protein present bound to pyocin S2 residues 556–688. The structure was manually rebuilt to match the sequence of pyocin S2 and refined iteratively using REFMAC5 [42] and Coot 0.7 [43].

### Crystal structure determination of the pyocin AP41 DNase domain

Crystallisation trials of pyocin AP41 DNase were performed against sparse screens at ≈ 60 mg ml^− 1^. Crystals spontaneously formed under condition 40 from Hampton Crystal Screen: 0.1 M trisodium citrate, 20% (v/v) 2-propanol and 20% (w/v) PEG 4000 (pH 5.6). Crystals were successfully reproduced and cyroprotected in 0.1 M trisodium citrate (pH 5.6), 22% (w/v) PEG 4000, 20% (v/v) 2-propanol and 20% (v/v) ethylene glycol. Diffraction and fluorescence data were collected from a single cryocooled crystal at beamline I04 of Diamond Light Source (United Kingdom).

Fluorescence data were analysed by PyMCA [44]. Diffraction data were integrated with XDS [38] and scaled and merged using Aimless [39]. The structure was solved by molecular replacement using Phaser [41] with the DNase domain of ColE9 as a search model [20]. Manual model building was performed in Coot 0.7 [43] and the structure was refined using PHENIX 1.9 [45].

### Crystal structure determination of the pyocin AP41 DNase–ImAP41 complex

Crystallisation of the pyocin AP41 DNase–ImAP41 complex was performed in sparse matrices. Complex crystals formed in 40% (v/v) Jeffamine D2000 and 0.1 M Hepes (pH 6.5) from the MIDAS screen after 16 weeks at 18 °C. Diffraction data were collected from a single cryocooled crystal at beamline I02 of Diamond Light Source (United Kingdom). The crystals diffracted X-rays to ≈ 1.8 Å; however, multiple lattices were present. The diffraction data were processed using XDS [38] to identify a single lattice that was subsequently integrated in XDS [38] and scaled and merged using Aimless [39]. A molecular replacement solution was found using Phaser [41] with the pyocin AP41 DNase domain as a search model. ImAP41 was built in Coot 0.7 [43] using Im9 as a guide and changing residues as required and the structure was refined using PHENIX 1.9 [45].

### Structure analysis

Protein structures were analysed using Coot [43] and PyMOL [46] molecular graphics programs. Buried surface area was calculated using PISA [47] and shape complementarity was calculated using Rosetta and RosettaScripts [48,49]. Figures of protein structures were created using PyMOL [46].

### Endonuclease activity assay

Pyocin AP41 DNase was incubated in 50 mM Tris (pH 7.5), 10 mM ethylenediaminetetraacetic acid (EDTA) for 1 h at 25 °C to remove bound metal ions. Protein was dialysed overnight against 50 mM Tris (pH 7.5) with two buffer changes. For the plasmid nicking assay, 1 μg pET21a plasmid DNA was added to 0.9 μM pyocin AP41 DNase in the presence of 10 mM NiCl_2_ and/or ImAP41 and the reactions were incubated at 25 °C. Metal dependence was tested by performing the reactions in the presence of 10 mM MgSO_4_ and 10 mM NiSO_4_ or ZnSO_4_. Reactions were stopped by the addition of 5 mM EDTA. Endonuclease activity was analysed by the presence of nicked plasmid DNA on a SybrSafe stained 0.6% agarose gel in TBE buffer.

### Accession number

Coordinates and structure factors have been deposited in the Protein Data Bank with accession numbers 4UHQ for the AP41 DNase domain, 4UHP for the AP41 DNase–ImAP41 complex and 4QKO for the S2 DNase–ImS2 complex.

The following is supplementary related to this article.

Supplemental Fig. 1 Probability of positive selection within colicin and pyocin DNase domains

## Figures and Tables

**Table 1:** X-ray diffraction and structure refinement statistics.

	PyoS2 DNase–ImS2	PyoAP41 DNase–ImAP41	AP41 DNase
*Data collection*^a^			
Space group	*P*2_1_2_1_2_1_	*P*1	*P*4_1_2_1_2
Cell dimensions			
*a*, *b*, *c* (Å)	65.39, 114.42, 120.22	36.60, 75.62, 83.18	100.57, 100.57, 71.53
α, β, γ (°)	90, 90, 90	79.21, 78.04, 77.00	90, 90, 90
Resolution (Å)	82.88–1.80 (1.83–1.80)	40.24–2.00 (2.05–2.00)	44.98–1.50 (1.53–1.50)
Solvent content (%)	56	42	59
No. of unique observations	83,310 (4379)	55,279 (4067)	59,171 (2870)
Multiplicity	4.2 (4.0)	1.8 (1.8)	13.0 (11.8)
Completeness (%)	99.1 (96.4)	97.2 (96.2)	100 (99.3)
*R*_merge_	0.062 (0.234)	0.176 (0.938)	0.056 (1.700)
*R*_pim_^b^	0.048 (0.190)	0.176 (0.938)	0.023 (0.728)
Mean *I*/sigma (*I*)	10.2 (2.8)	4.7 (2.7)	22.0 (1.6)
CC_1/2_^c^	N/A	0.993 (0.733)	1.00 (0.539)

*Refinement statistics*			
*R*_work_/*R*_free_	0.174/0.215	0.189/0.233	0.173/0.191
No. of non-hydrogen atoms	7828	7581	2313
RMSD of bond lengths (Å)	0.020	0.002	0.013
RMSD of bond angles (°)	1.78	0.640	1.46
No. of waters	815	596	135
Mean/Wilson plot *B*-value (Å^2^)	29.38/33.8	32.0/17.4	30.8/20.7
Ramachandran plot (%)^d^			
Favoured/allowed/outliers	98.1/1.9/0.0	98.5/1.5/0.0	100/0.0/0.0
PDB identifier	4QKO	4UHP	4UHQ

a Values in parentheses refer to the highest-resolution shell.

b *R*_pim_ = ∑*_hkl_*[1/(*N* − 1)]^1/2^∑*_i_*|*I_i_*(*hkl*) − 〈*I*(*hkl*)〉|/∑*_hkl_*∑*_i_I_i_*(*hkl*).

c CC_1/2_ is half-dataset correlation coefficient [46].

d Percentages of residues in favoured/allowed regions were calculated by the program MolProbity [47].

**Table 2:** Intermolecular interaction statistics in DNase–Im complexes.

Complex	Buried surface area (Å^2^)	Direct salt bridges/hydrogen bonds	Buried waters	Shape complementarity^a^	PDB code
PyoS2–ImS2	1590.5	11/20	10	0.70	4QKO^b^
PyoAP41–ImAP41	1744.0	8/16	13	0.74	4UHP^b^
ColE2–Im2	1692.0	12/18	13	0.73	3U43
ColE7–Im7	1432.4	7/16	10	0.70	1MZ8
ColE9–Im9	1533.7	5/13	7	0.72	1EMV

a As per Ref. [48].

b This work.
