# Supplementary figures and images for: Structures of the Ultra-High-Affinity Protein–Protein Complexes of Pyocins S2 and AP41 and Their Cognate Immunity Proteins from Pseudomonas aeruginosa
Source: J Mol Biol. 2015 Aug 28;427(17):2852–66. doi: 10.1016/j.jmb.2015.07.014 (PMC4548480; doi:10.1016/j.jmb.2015.07.014)

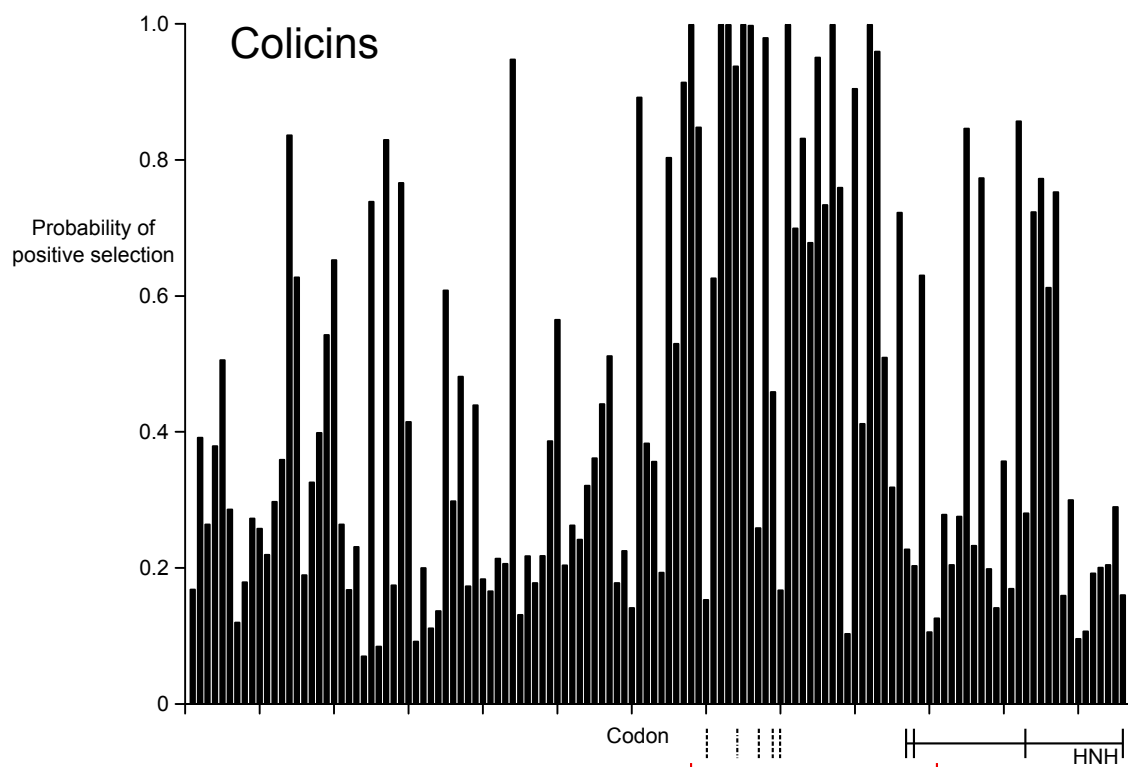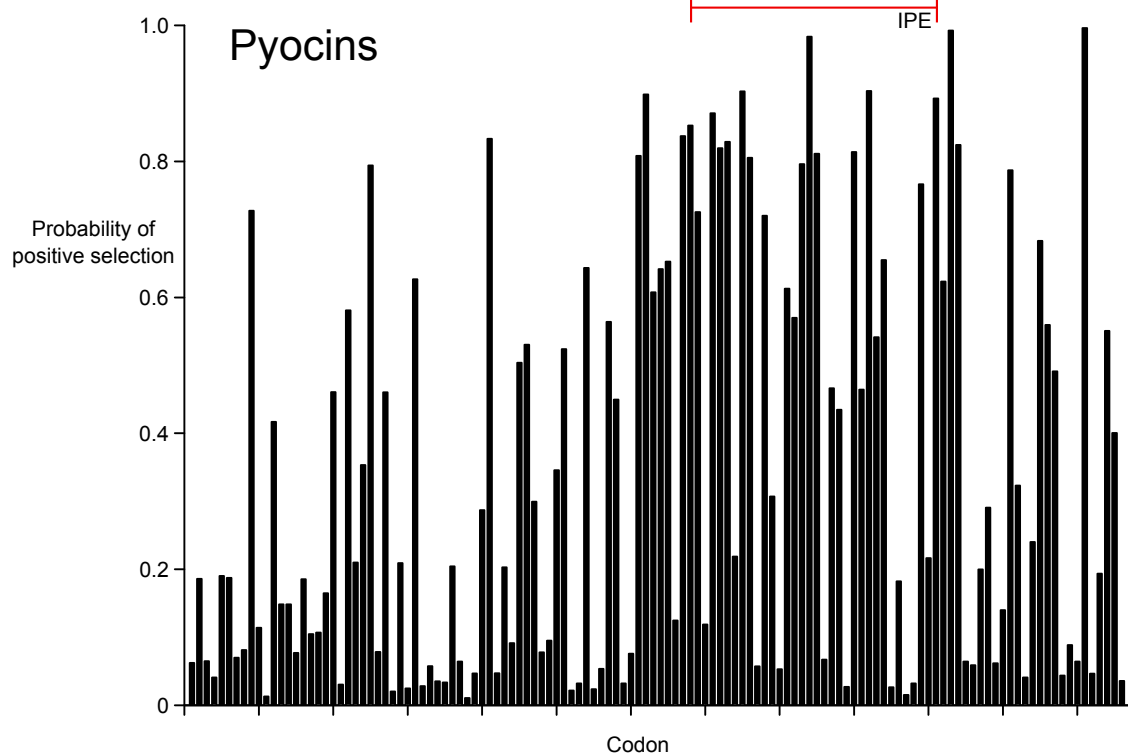

Supplement: Supplemental Fig. 1 — Probability of positive selection within colicin and pyocin DNase domains [file mmc1.pdf]
